# COMBAT-C: COntrol of Major Bleeding by Application of Tourniquets over Clothing

**DOI:** 10.1186/s12873-024-01005-x

**Published:** 2024-05-30

**Authors:** Raimund Lechner, Amelie Oberst, Yannick Beres, Benjamin Mayer, Martin Kulla, Björn Hossfeld, Holger Gässler

**Affiliations:** 1 Department of Anesthesiology, Intensive Care Medicine, Emergency Medicine and Pain Therapy, Bundeswehr Hospital Ulm, Oberer Eselsberg 40, 89081 Ulm, Germany; 2 Medical Service Police Baden-Württemberg, Stuttgart, Germany; 3 Institute for Epidemiology and Medical Biometry, Ulm University, Ulm, Germany

**Keywords:** Combat application tourniquet, SAM extremity tourniquet, SOF tactical tourniquet – wide, Critical bleeding, Hapmed tourniquet trainer

## Abstract

**Introduction:**

External bleeding is the leading cause of preventable trauma-related death. In certain circumstances, tourniquet application over clothing may be necessary. Therefore, the aim of this study was to assess the effectiveness of tourniquets over different clothing setups.

**Methods:**

Three windlass tourniquets (CAT, SAMXT, SOFTT-W) were applied over nine different clothing setups and without clothing on the Hapmed™ Tourniquet Trainer. We compared each tourniquet in each clothing setup to the tourniquet trainer that was not dressed, and we compared the three tourniquets within each clothing setup concerning blood loss, applied pressure and application time. Regression analysis of the effect of thickness, mean weight, mean deformation, application time, and applied pressure on blood loss was performed.

**Results:**

Although blood loss was significantly greater in the CAT and SAMXT tourniquets when they were applied over leather motorcycle trousers, the overall findings showed that the clothing setups significantly reduced or did not affect blood loss. The mean blood loss was the lowest with CAT and the highest with SOFTT-W. The measured mean pressures were lower than 180 mmHg in four out of nine clothing setups with SOFTT-W, but CAT and SAMXT always exceeded this threshold. CAT had the fastest application time. Blood loss was significantly influenced by applied pressure and application time but was influenced to a far lesser degree by clothing parameters.

**Conclusion:**

The effects of the clothing setups were of little clinical relevance, except for leather motorcycle trousers. The effects of rugged protective equipment, e.g., hazard suits, are conceivable and need to be tested for specific garments with the tourniquet intended for use. No clothing parameter for predicting tourniquet effectiveness could be identified.

## Introduction

Bleeding has been the leading preventable cause of death in both military and civilian trauma cases for decades [1–5]. Depending on the patient population, between 13.5% and 39% of cases of potentially survivable hemorrhage can be treated with an extremity tourniquet [1, 3]. According to all the established trauma guidelines, in cases where external bleeding cannot be stopped by other methods or in time-critical situations, tourniquet application is recommended for the management of critical limb hemorrhage [2, 6–8]. A tourniquet is typically applied directly to the skin 2–3 inches (approximately the width of the hand) proximal to the site of injury [7, 9]. However, the tourniquet should be temporarily applied over clothing in situations of direct threat and time pressure where undressing is not possible or not appropriate, such as mass casualty incidents; chemical, biological, radiological, and nuclear threats; extreme environmental conditions (cold, high altitude, wetness, and high wind speeds); and scenarios where victims are inaccessible because they are trapped, buried, or in the dark [7, 10–13].

The effectiveness of tourniquets over clothing has been analysed for only a few types of tourniquets and a few clothing setups. In general, the application of tourniquets over clothing appears to be possible but is a poorly studied subject, and studies thus far often lack objective parameters such as estimated blood loss and tourniquet pressure [10, 13–17]. Furthermore, there are no clear objective textile parameters that can be used to predict the effectiveness of applying tourniquets over clothing. The objective of this study was to evaluate the effectiveness of three commonly used windlass tourniquets applied over various military and civilian clothing layers and to identify possible textile parameters that may predict the effectiveness of tourniquet application over clothing.

## Methods

### Ethical considerations

The ethics committee of the University of Ulm waived the need for ethical approval due to the simulator-based design of this study, in accordance with paragraph 15 of the Professional Code of Conduct of the Baden-Württemberg Medical Association (“Landesärztekammer”).

### Experimental setup and training

**Fig. 1:**
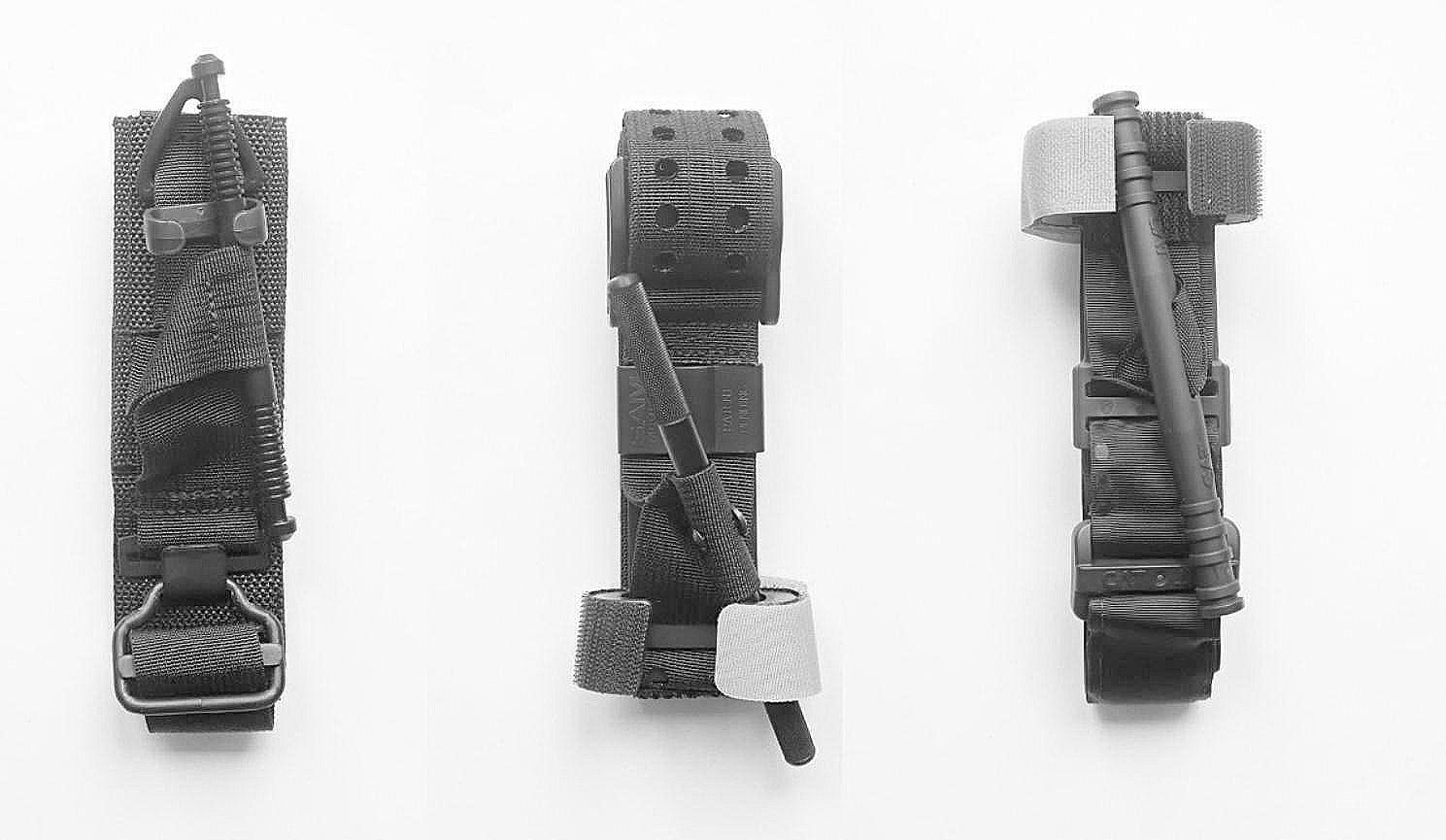
The used tourniquets. From left to right: The Combat Application Tourniquet^®^ Generation 7 (C•A•T Resources LLC, Rock Hill, SC, USA; CAT), the SAM^®^ Extremity Tourniquet (Sam Medical, Tualatin, OR, USA; SAMXT), and the SOF^®^ Tactical Tourniquet Wide Generation 4 (TacMed Solutions, Anderson, SC, USA; SOFTT-W)

The Combat Application Tourniquet® Generation 7 (C•A•T Resources LLC, Rock Hill, SC, USA; CAT), the SAM® Extremity Tourniquet (Sam Medical, Tualatin, OR, USA; SAMXT), and the SOF® Tactical Tourniquet Wide Generation 4 (TacMed Solutions, Anderson, SC, USA; SOFTT-W) were analysed on a Hapmed™ tourniquet trainer (leg number 0082, v2.17.25; CHI Systems, Plymouth Meeting, PA, USA) (Fig. 1). The application settings included clothing the manikin in different types of military and civilian trousers (Table 1) in various combinations (Tables 2, 3 and 4). The tourniquet trainer represents a thigh with an above-knee amputation injury. It has an in-built system for the measurement of directly applied pressure and time and a proprietary algorithm for the calculation of blood loss. It has been used in several tourniquet evaluation trials [18–21]. The scenario we chose involves bleeding within 2 min and requires the placement of a tourniquet on the proximal limb via a “high and tight” approach [22].

**Table 1:** Characteristics of the garments used for the clothing setups with standard deviation

Trousers	Thickness [mm]	Mass per unit area [g/m^2^]	Deformation [mm/kPa]
Bundeswehr combat trousers	0.50 ± 0.02	23.42 ± 0.10	0.03 ± 0.00
Bundeswehr rain trousers	0.40 ± 0.01	17.43 ± 0.02	0.02 ± 0.00
Bundeswehr insulation trousers	3.34 ± 0.07	32.69 ± 0.97	0.39 ± 0.03
Bundeswehr insulation sublayer	2.91 ± 0.17	26.48 ± 0.36	0.10 ± 0.01
Bundeswehr Overgarment	1.56 ± 0.02	49.53 ± 0.26	0.07 ± 0.00
Bundeswehr tropical trousers	0.50 ± 0.01	22.74 ± 0.10	0.03 ± 0.00
Cut Protection Trousers	8.12 ± 0.18	171.80 ± 4.40	0.22 ± 0.01
Synthetic motorcycle trousers	0.67 ± 0.09	34.54 ± 1.57	0.07 ± 0.03
Leather motorcycle trousers	2.11 ± 0.07	104.46 ± 9.23	0.05 ± 0.01

**Table 2:** Blood loss [ml]

	CAT	SAMXT	SOFTT-W	CAT vs. SAMXT	CAT vs. SOFTT-W	SAMXT vs. SOFTT-W
Undressed	99 ± 17	141 ± 28	177 ± 71	*	*	*
Bundeswehr combat trousers	110 ± 23	105 ± 27 *	148 ± 42 *		*	*
Bundeswehr insulation sublayer + Bundeswehr combat trousers	104 ± 19	121 ± 18	168 ± 38		*	*
Bundeswehr combat trousers + Bundeswehr rain trousers	113 ± 24	104 ± 30 *	135 ± 32 *			*
Bundeswehr combat trousers + Bundeswehr insulation trousers	98 ± 9	105 ± 23 *	134 ± 22 *		*	*
Bundeswehr combat trousers + Bundeswehr insulation trousers + Bundeswehr rain trousers	116 ± 21	133 ± 28	174 ± 30		*	*
Bundeswehr tropical trousers + Bundeswehr Overgarment	110 ± 18	151 ± 62	145 ± 22 *	*	*	
Cut protection trousers	91 ± 14	151 ± 44	139 ± 26 *	*	*	
Synthetic motorcycle trousers	95 ± 26	150 ± 40	135 ± 40 *	*	*	
Leather motorcycle trousers	135 ± 25 *	174 ± 70 *	176 ± 53	*	*	

Blood loss corresponding to each tourniquet for each of the nine clothing setups compared to the undressed setting and comparison of the three tourniquets within each clothing setup

The data are presented as the mean ± standard deviation

CAT: Combat Application Tourniquet® Generation 7; SAMXT: SAM® Extremity Tourniquet; SOFTT-W: SOF® Tactical Tourniquet Wide Generation 4

*Significant at *p* < 0.05

**Table 3:** Applied pressure [mmHg]

	CAT	SAMXT	SOFTT-W	CAT vs. SAMXT	CAT vs. SOFTT-W	SAMXT vs. SOFTT-W
Undressed	494 ± 140	442 ± 105	240 ± 62	*	*	*
Bundeswehr combat trousers	503 ± 89	409 ± 84	297 ± 67 *	*	*	*
Bundeswehr insulation sublayer + Bundeswehr combat trousers	449 ± 60	463 ± 88	127 ± 62 *		*	*
Bundeswehr combat trousers + Bundeswehr rain trousers	528 ± 80	477 ± 89	182 ± 94 *		*	*
Bundeswehr combat trousers + Bundeswehr insulation trousers	476 ± 58	547 ± 95 *	262 ± 84	*	*	*
Bundeswehr combat trousers + Bundeswehr insulation trousers + Bundeswehr rain trousers	530 ± 97	389 ± 67 *	160 ± 49 *	*	*	*
Bundeswehr tropical trousers + Bundeswehr Overgarment	537 ± 100	540 ± 112 *	293 ± 72 *		*	*
Cut protection trousers	512 ± 75	386 ± 81 *	157 ± 62 *	*	*	*
Synthetic motorcycle trousers	461 ± 78	366 ± 91 *	211 ± 104	*	*	*
Leather motorcycle trousers	318 ± 75 *	316 ± 73 *	115 ± 68 *		*	*

Applied pressure corresponding to each tourniquet for each of the nine clothing setups compared to the undressed setting and comparison of the three tourniquets within each clothing setup

The data are presented as the mean ± standard deviation

CAT: Combat Application Tourniquet® Generation 7; SAMXT: SAM® Extremity Tourniquet; SOFTT-W: SOF® Tactical Tourniquet Wide Generation 4

*Significant at *p* < 0.05

**Table 4:** Application time [seconds]

	CAT	SAMXT	SOFTT-W	CAT vs. SAMXT	CAT vs. SOFTT-W	SAMXT vs. SOFTT-W
Undressed	24 ± 4	34 ± 6	34 ± 13	*	*	
Bundeswehr combat trousers	25 ± 5	31 ± 8	34 ± 13	*	*	
Bundeswehr insulation sublayer + Bundeswehr combat trousers	24 ± 4	32 ± 9	27 ± 8 *	*		*
Bundeswehr combat trousers + Bundeswehr rain trousers	27 ± 5	25 ± 6 *	30 ± 10			
Bundeswehr combat trousers + Bundeswehr insulation trousers	20 ± 3	25 ± 5 *	24 ± 7 *			
Bundeswehr combat trousers + Bundeswehr insulation trousers + Bundeswehr rain trousers	31 ± 7 *	33 ± 10	25 ± 5 *		*	*
Bundeswehr tropical trousers + Bundeswehr Overgarment	29 ± 6 *	41 ± 15 *	38 ± 12	*	*	
Cut protection trousers	21 ± 3	36 ± 10	24 ± 4 *	*		*
Synthetic motorcycle trousers	23 ± 3	28 ± 7 *	27 ± 4 *	*		
Leather motorcycle trousers	25 ± 3	42 ± 13 *	30 ± 6	*	*	*

Application time corresponding to each tourniquet for each of the nine clothing setups compared to the undressed setting and comparison of the three tourniquets within each clothing setup

The data are presented as the mean ± standard deviation

CAT: Combat Application Tourniquet® Generation 7; SAMXT: SAM® Extremity Tourniquet; SOFTT-W: SOF® Tactical Tourniquet Wide Generation 4

*Significant at *p* < 0.05

**Fig. 2:**
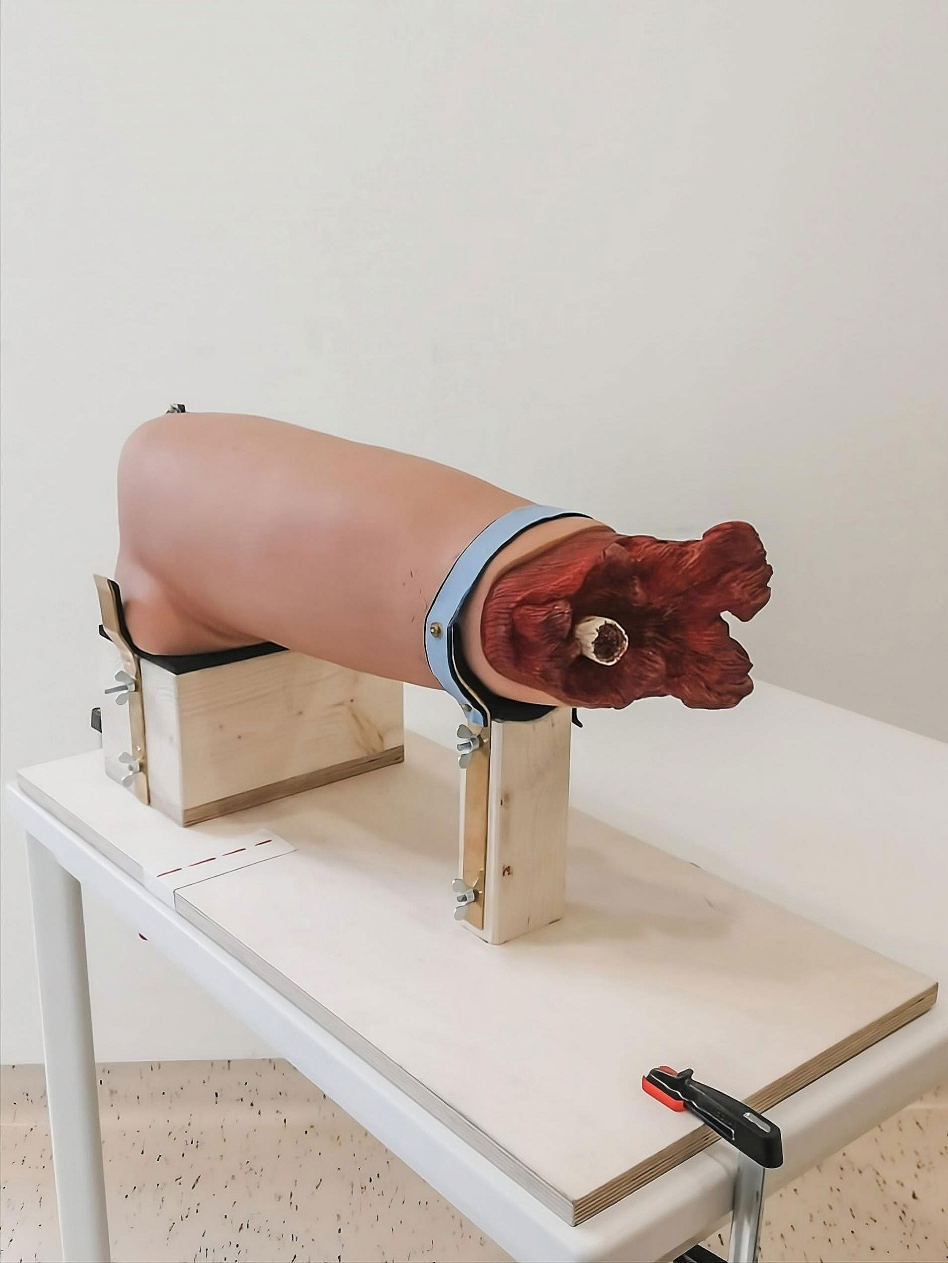
The hapmed^™^ tourniquet trainer mounted in a custom-made apparatus to ensure standardized application. The red dotted line marks the position of the tourniquet application

Two experienced providers (AO, YB) gave informed consent to participate in the study and were trained in the use of the tourniquet models until they could achieve correct and rapid application. Both providers applied each tourniquet with both hands 10 times in each clothing setup. They were blinded to the display of the tourniquet trainer. The tourniquet trainer was mounted in a custom-made apparatus to ensure standardized application (Fig. 2). The 2 providers stood 1 m away from the table with the tourniquet trainer (marking on the floor) and held the opened tourniquet in one hand. At a signal from the timekeeper, they applied the tourniquet as quickly as possible and with the maximum possible tightness at the height of the red dotted line. The time was stopped as soon as the windlass was secured.

### Measurement of variables

The pressure applied, blood loss, and application time were recorded. Effectiveness was defined as pressure greater than 180 mmHg according to the recommendations of the Committee on Tactical Combat Casualty Care (CoTCCC) [23]. The following three parameters were measured five times for each type of clothing, and the mean value was calculated: thickness, according to ISO 5084:1996 (DMT 100 with S229 gauge; Sylvac, Yverdon, Switzerland); mass per unit area, according to DIN EN 12127:1997 (Weighing Balance Discovery DV 215 CDM; Ohaus, Parsippany, NJ, USA); and deformation of each piece of clothing (testControl II; ZwickRoell, Ulm, Germany) (Table 1) [24, 25]. These measurements and calculations were performed at the Bundeswehr Research Institute for Materials, Fuels and Lubricants.

### Statistical analysis

We compared each tourniquet in each clothing setup to the tourniquet trainer that was not dressed, and we compared the three tourniquets within each clothing setup. A mixed linear regression analysis was used because of the repeated-measures design of the experiment (SAS/STAT, Heidelberg, Germany). Linear contrasts were applied for pairwise comparisons of interest. The model included clothing setup, tourniquet model, and their interaction as fixed variables, with the provider considered a covariate. A random intercept was assumed for each provider. For all tested clothing setups and all single-layer clothing setups, we performed multivariable linear regression of blood loss, applied pressure, time, total thickness, mean mass per unit area, and mean deformation (SPSS/IBM, Armonk, NY, USA). The explorative type 1 error level was set to 5% (two-sided value).

## Results

### Provider-dependent differences in tourniquet application variables

Each tourniquet was applied 10 times by each provider over each clothing setup. This amounted to a total of 600 applications. With a type one error of 5%, a statistically significant difference was found between providers for blood loss but not for pressure applied or application time.

### Blood loss according to tourniquet type and clothing setup

Blood loss was significantly lower than that in the undressed setting in some clothing setups, while it was not significantly affected in other clothing setups. The only exception was the setting with leather motorcycle trousers, as blood loss compared to that in the undressed setting was significantly greater with CAT and SAMXT. With the application of SOFTT-W to leather motorcycle trousers, blood loss was similar to that in the undressed setting but was significantly greater than that with CAT and similar to that with SAMXT. The mean blood loss across all clothing setups was the lowest for CAT and the highest for SOFTT-W (Table 2).

### Application pressure according to tourniquet type and clothing setup

For SOFTT-W, the mean application pressure remained below the cut-off of 180 for the clothing combinations of *insulation sublayer + combat trousers*, *combat trousers + insulation trousers + rain trousers*, *cut-protection trousers*, and *leather motorcycle trousers*. All the other measurements were above the cut-off of 180 mmHg. The pressure generated was significantly lower for the SOFTT-W than for the CAT and SAMXT in all the clothing setups. In the case of the leather motorcycle trousers, the pressure was significantly lower in the case of all three tourniquets than in the case of the undressed setting. For the other clothing setups, the differences compared to those in the undressed setting were significant in some cases, but the differences were not homogeneous across all tourniquets (Table 3).

### Application time according to tourniquet type and clothing setup

In the case of SOFTT-W, the application time was significantly unchanged or significantly reduced for all clothing setups compared to the undressed setting, while it was significantly longer in the case of SAMXT application to the *tropical trousers + overgarment combination* and *leather motorcycle trousers* setups and CAT application to the *combat trousers + insulation trousers + rain trousers* and the *tropical trousers + overgarment* setups. The CAT tourniquet had the most consistent application times and, with two exceptions, also had the fastest application time (Table 4).

### Regression analysis of factors associated with blood loss

Regression analysis of the clothing setups showed that applied pressure and application time were strongly associated with blood loss for all the tourniquets tested. Among the tested textile characteristics, only the mean deformation was found to be significant in the case of SAMXT. Furthermore, no multicollinearity was observed (Table 5).

**Table 5:** Regression analysis on blood loss for all the clothing setups

CAT			
explanatory variables	coefficients	standard error	*p*
bloodloss (constant)	91.25		
applied pressure	-0.08	0.016	< 0.001
time	2.34	0.29	< 0.001
thickness	-1.52	1.68	0.37
mean weight	0.06	0.05	0.24
mean deformation	-4.37	42.39	0.92
R^2^	0.36		
R^2^adjusted	0.35		
F (df = 5; 174)	19.97		
**SAMXT**			
**explanatory variables**	**coefficients**	**standard error**	**p**
bloodloss (constant)	103.78		
applied pressure	-0.15	0.023	< 0.001
time	2.78	0.23	< 0.001
thickness	-4.67	2.68	0.08
mean weight	0.07	0.08	0.39
mean deformation	148.7	67.74	0.03
R^2^	0.58		
R^2^adjusted	0.56		
F (df = 5; 174)	47.08		
**SOFTT-W**			
**explanatory variables**	**coefficients**	**standard error**	**p**
bloodloss (constant)	115.75		
applied pressure	-0.19	0.027	< 0.001
time	2.42	0.29	< 0.001
thickness	2.39	2.68	0.38
mean weight	-0.14	0.07	0.05
mean deformation	36.82	69.85	0.60
R^2^	0.37		
R^2^adjusted	0.36		
F (df = 5; 174)	20.76		

Regression analysis of the effects of application time, applied pressure, and textile characteristics (total thickness, mean mass per unit area, and mean deformation) on blood loss for all the clothing setups

CAT: Combat Application Tourniquet^®^ Generation 7; SAMXT: SAM^®^ Extremity Tourniquet; SOFTT-W: SOF^®^ Tactical Tourniquet Wide Generation 4

Regression analysis of the single-layer clothing setups (*combat trousers*, *cut-protection trousers*, *synthetic motorcycle trousers*, and *leather motorcycle trousers*) showed that applied pressure and application time were strongly associated with blood loss for all the tourniquets tested, without violation of the multicollinearity assumption. The tested textile characteristics were significant for the CAT tourniquet in terms of mean deformation and for the SAMXT tourniquet in terms of all the tested textile parameters. However, the multicollinearity assumption was consistently violated for all three clothing parameters (thickness, mean weight, and mean deformation) in all the tested tourniquets (Table 6).

**Table 6:** Regression analysis on blood loss for single-layer clothing

CAT			
explanatory variables	coefficients	standard error	*p*
bloodloss (constant)	87.16		
applied pressure	-0.06	0.03	0.046
time	2.38	0.70	0.001
thickness	5.58	7.38	0.452
weight	0.14	0.21	0.512
deformation	-378.83	183.26	0.042
R^2^	0.48		
R^2^adjusted	0.44		
F (df = 5; 74)	13.47		
**SAMXT**			
**explanatory variables**	**coefficients**	**standard error**	**p**
bloodloss (constant)	66.86		
applied pressure	-0.21	0.05	< 0.001
time	2.99	0.43	< 0.001
thickness	-40.86	12.12	0.001
weight	0.79	0.33	< 0.001
deformation	1148.27	308.34	0.02
R^2^	0.57		
R^2^adjusted	0.54		
F (df = 5; 74)	19.571		
**SOFTT-W**			
**explanatory variables**	**coefficients**	**standard error**	**p**
bloodloss (constant)	126.21		
applied pressure	-0.22	0.05	< 0.001
time	2.74	0.48	< 0.001
thickness	7.74	11.81	0.51
weight	− 0.09	0.33	0.78
deformation	− 298.64	294.27	0.31
R^2^	0.50		
R^2^adjusted	0.47		
F (df = 5; 74)	15.01		

Regression analysis of the effect of application time, applied pressure, and textile characteristics (total thickness, mean mass per unit area, and mean deformation) on blood loss for single-layer clothing

CAT: Combat Application Tourniquet^®^ Generation 7; SAMXT: SAM^®^ Extremity Tourniquet; SOFTT-W: SOF^®^ Tactical Tourniquet Wide Generation 4

## Discussion

This study examines the effectiveness of three windlass tourniquets (CAT, SAMXT, and SOFTT-W) when applied over different clothing setups. The CAT tourniquet had the lowest blood loss for all the clothing setups, followed by SAMXT and SOFTT-W. An average pressure of more than 180 mmHg was achieved with the CAT and the SAMXT tourniquets in all clothing setups, but this could not be achieved with the SOFTT-W tourniquet in four out of nine clothing setups. On average, the pressure achieved with the SOFTT-W was significantly lower than that achieved with CAT and SAMXT. Finally, CAT had the fastest application time. Overall, according to our data, CAT seems to be the most effective of the three tourniquets tested for application over clothing.

Tourniquets can be roughly divided into elastic band tourniquets, ratcheting tourniquets, pneumatic tourniquets, and windlass tourniquets, of which the windlass tourniquet type is the most commonly used [23]. Our aim was to analyze the most used tourniquet type in detail, which is why we have not included any other tourniquet types. Although all the tourniquets tested here are recommended by the CoTCCC in general, there are certain differences between them [23]. CAT is the standard tourniquet used in most limb tourniquet studies, is the standard tourniquet used by most military forces and is, in our opinion, to be regarded as a kind of benchmark [23]. The SAMXT is a more recent tourniquet model, characterized by a special buckle design, which is intended to reduce slack. The windlass and buckles of the SOFTT-W are made of metal, in contrast to most tourniquets. The CAT and the SOFTT were already recommended by the CoTCCC before 2019, so they are widely used, and a lot of experience is available. Notably, SOFTT-W Generation 4, which was used in this study, was not evaluated by the CoTCCC, and only the preceding model (SOFTT-W Generation 3) was recommended. The Generation 4 tourniquet is characterized by a novel windlass and quick compression buckle design, the addition of a retention clip, and the use of reinforced and more durable polyester material [26]. We are not aware of any studies that have compared SOFTT-W Generation 3 and 4. We found only one other study that evaluated the SOFTT-W Generation 4 model [27], and the results showed that SOFTT-W Generation 4 had significantly greater failure rates (based on Doppler signal measurements) and longer application times than did CAT. In addition, it should be noted that SOFTT-W Generation 5 is now commercially available, with a newly designed buckle and a slack indicator. The SAMXT tourniquet had a slight modification at the buckle stitch in 2018, which, due to its insignificance, is unlikely to affect the comparability of published study results [28]. The discussion of the results must be based on these limitations, especially since some studies do not explicitly mention which generation of the tourniquet was used.

Blood loss is mainly a function of pressure and time (Tables 5 and 6). An increase in blood loss can be explained by prolonged application time, insufficient maximum pressure, or insufficient increase in pressure over time. An increase in pressure over time was not recorded in this experiment, but this difference may explain the lower blood loss observed in patients with lower maximum pressure or prolonged application time. That is, when the applied pressure is too low, there is a risk of increased blood loss. There were significant differences in pressure between the CAT and SAMXT tourniquets, but the applied pressure was always clearly above 180 mmHg, as recommended by the CoTCCC [23]. Thus, acute hemorrhage can most likely be controlled by both tourniquets. However, this was not the case with the SOFTT-W tourniquet, which had significantly lower applied pressures than did the CAT and the SAMXT tourniquets, as well as significantly greater blood loss in most clothing setups. Low tourniquet pressure may intensify bleeding by preventing venous flow while maintaining arterial flow, which is comparable to venous stasis for blood sampling. Venous stasis is a relevant variable in the real-life setting, but it is not clear whether it is considered in the algorithm of the Hapmed^™^ trainer. Thus, the low pressure applied with the SOFTT-W tourniquet may have led to underestimation of blood loss.

According to the CoTCCC recommendations, all three tourniquets met the criteria for application pressure, but the SOFTT-W had lower application pressure in our study [23]. In line with our findings, more recent studies conducted after the CoTCCC recommendations reported differences between the CAT, the SAMXT, and the SOFTT-W (Generation 3) tourniquets; for example, Katsnelson et al. found only minor pressure differences between the CAT and SAMXT but significantly lower pressure with the SOFTT-W [19]. However, they did not report any significant differences with regard to blood loss [19]. In contrast to their findings, high rebleeding rates were reported for the SOFTT-W tourniquet in another manikin study, which may be related to the low applied pressure [29]. In line with these findings, Wall et al. also reported that SOFTT-W generation 3 had lower application pressure than CAT, which was attributed to the inability to continue turning the windlass and secure it in the unique Tri-Ring Lock^™^ of the tourniquet after a high number of turns of the windlass [14].

A high tourniquet pressure contributes to iatrogenic injury, which is why the CoTCCC considers pressures up to 500 mmHg to be the optimal upper occlusion pressure [23, 30]. Pressures above 500 mmHg were achieved primarily via CAT over various clothing setups in our study. However, higher pressures presumably cause greater patient discomfort. In fact, other studies on tourniquet application over clothing have shown that CAT was more painful than was SOFTT-W, although applying it over clothing was generally found to be more comfortable than applying it on bare skin [14, 16]. In addition, self-reported pain was also lower with multilayer clothing than with single-layer clothing; this difference was attributed to reduced skin pinching, which is thought to be responsible for tourniquet-induced pain, especially in the early period after application [13]. Anecdotally, pain is a common cause of inadvertent opening of the tourniquet by the injured person. Since application over clothing is always a short-term bridging measure, achieving sufficient occlusion pressure is more relevant than not exceeding the maximum pressure. Thus, in our opinion, the tourniquet with the highest applied pressure, i.e., the CAT tourniquet, is the most effective for application in clothing.

In the present study, the application time tended to decrease with the number of applications, despite the high level of previous experience of the users. This was especially evident with the SOFTT-W, which showed that the application time can be improved through a high number of repetitive tourniquet applications. We were unable to identify studies that reported a similar number of applications to our study (100 applications for each tourniquet model per user); however, in general, training volume is known to reduce application time without affecting tourniquet pressure [18]. Because blood loss is a function of pressure over time, blood loss may have decreased with increasing training volume, which may have resulted in bias in the results. The CoTCCC considers an occlusion time of less than 60 s and 90 s to indicate successful completion of tourniquet application [23]. The mean application times in this study were consistently less than 60 s, which means that the applications were successfully completed.

In the tested clothing setups, blood loss was generally lower than that in the undressed setting, but the difference was only partially significant. In the case of the leather motorcycle trousers, pressure was significantly lower in the case of all tourniquets, and significantly greater blood loss occurred with CAT and SAMXT. In addition, with SAMXT, the application time was significantly longer with the *leather motorcycle trousers* and the *tropical trousers + overgarment* setting. The SAMXT tourniquet has a unique autolock mechanism with prongs on the buckle and a strap with holes to reduce the slack of the compression strap and create sufficient pretightening before the windlass is used [19]. The difficulty in efficiently applying this locking mechanism to particular types of clothing (e.g., smooth leather and smooth overgarment) could be responsible for the significantly longer application times in our study. Prolonged application time may result in increased blood loss, which was significant in the case of the leather motorcycle trousers. In line with our findings, the CoTCCC evaluation also revealed that SAMXT achieved the lowest value among the tourniquets studied here in terms of application time and was only rated as acceptable [23]. In other studies, with different clothing setups, the application of CAT was significantly faster than that of SOFTT-W Generation 3, but the application time was not significantly altered by different clothing setups [13, 16]. Thus, it appears that the application time is more dependent on the tourniquet model than on the clothing setup [16]. Thus, in terms of the mean application time, CAT is the fastest on average and, therefore, the most effective, followed by SOFTT-W and SAMXT.

To our knowledge, cut-protection trousers and motorcycle trousers, especially those made of leather, have not yet been tested in terms of tourniquet application, whereas scrubs, tights, different uniform trousers, rain trousers, chemical/biological/radiological/nuclear protective equipment, and different winter (warfare) configurations have been analysed [10, 13–16, 31]. The outcome parameters varied and included subjective user ratings, Doppler and pulse measurements, pulse oximetry, and measured tourniquet pressures. However, (calculated) blood loss associated with the application of tourniquets over clothing has not yet been assessed. Previous studies were conducted partly on simulators and partly on humans, and tourniquet use over clothing was generally judged to be sufficient. The measured tourniquet pressures were found to be greater with clothing than without clothing, but the pressures tended to be lower than the values recorded in our analysis [14]. This difference is probably attributable to the difference in the experimental design used for pressure assessment (tourniquet trainer versus neonatal blood pressure cuff).

By measuring thickness, mass per unit area, and deformation, we attempted to identify objective textile parameters related to tourniquet effectiveness. These clothing parameters have not been previously evaluated. However, these parameters influenced blood loss to a far lesser degree than did the tourniquet model, applied pressure, and application time (Tables 5 and 6). To choose realistic scenarios, some of our setups included several clothing layers on top of each other. However, these setups might have been affected by the effect of each layer on the other; for example, the friction between the layers may have limited the validity of the regression analysis of the textile parameters for the clothing combinations. Therefore, we performed a regression analysis with the single-layer clothing setups. Although the textile parameters resulted in significant changes, no valid statement can be made regarding which parameter is most responsible for the changes due to violation of the multicollinearity assumption. The SAMXT tourniquet was most clearly influenced by the clothing layers. However, the baseline values measured without clothing for blood loss, applied pressure, and time were sufficient (Tables 2, 3 and 4); therefore, the clinical relevance of the influence of clothing was modest. In contrast, for the SOFTT-W, the baseline values for the measurements without clothing were close to the critical limits. This means that hemostasis may not always be guaranteed with this tourniquet, even if the influence of clothing is modest. The CAT tourniquet was the least influenced by the clothing setup, and the baseline values without clothing were farthest from the critical limits. Various other textile parameters may influence tourniquet effectiveness, for example, bending stiffness in the warp and weft directions. These parameters could not be assessed in this study due to the thickness of the clothing. Ultimately, only leather was consistently associated with significantly lower applied pressure and increased blood loss. Overall, the differences caused by wearing different clothing types appear to be of little clinical relevance, except for leather motorcycle trousers.

To limit interuser variability and to detect only the effects of the clothing layers and the different tourniquet models, tourniquets were applied repetitively by only two providers, and a large user collective was not selected, analogous to other studies [15, 20, 32]. It is challenging to achieve a high and consistent training level across a group of participants in contrast to only two providers. In our opinion, a larger user group therefore harbors the risk that user effects instead of clothing and tourniquets characteristics significantly influence the results. By conducting the study with two providers, the influence of the tourniquet models and the clothing could be better identified, while any systematic error on the part of one provider could be excluded. Significant differences were observed between the providers only with regard to blood loss, but no significant difference was observed for application time or pressure. This finding indicates that the effectiveness of trained providers is primarily dependent on the tourniquet model and, In the case of application over clothing, to a far lesser degree on the clothing setup. In agreement with our findings, Wall et al. also reported that the hand strength of different providers had no effect on tourniquet pressure [14].

### Limitations

The main limitation of this study is that this was a simulator-based study in which measurements were not performed on humans. Therefore, vascular occlusion can be assumed based only on the surrogate parameter applied pressure. In addition, other relevant factors related to tourniquet application, such as patient discomfort and general patient characteristics (e.g., leg circumference), could not be assessed. As the blood loss calculation algorithm is proprietary, the magnitude of the impact of a decrease in blood pressure on blood loss (hemorrhagic shock) and, thus, the bleeding control achieved with the applied tourniquet pressure are not quantifiable.

Another limitation was that the feasibility of using a tourniquet device under laboratory conditions might be different from that in real-life scenarios with high stress levels, and the ability to apply a tourniquet may be different for providers who are not adequately trained. Finally, as extensively presented in the discussion, the data was only compiled by two providers and only three different windlass tourniquet models were analysed. Larger sample sizes and further tourniquet types, like elastic band tourniquets, ratcheting tourniquets or pneumatic tourniquets may be considered in follow-up studies.

## Conclusion

Among the three tourniquets tested in this study, the CAT tourniquet was the least affected by the clothing setups tested. For the SOFTT-W tourniquet, the measured pressures were lower than 180 mmHg in four out of nine clothing setups; thus, it is unclear whether adequate bleeding control can be achieved with this tourniquet. The effects of the clothing setups tested were of little clinical relevance in the case of CAT and SAMXT, apart from the leather motorcycle trousers. Overall, none of the clothing parameters tested in the present study were found to be predictors of tourniquet effectiveness. However, the effects of rugged protective equipment, e.g., hazard suits, are conceivable, and specific garments need to be tested with the tourniquet intended for use.

## Data Availability

The datasets used and/or analysed during the current study are available from the corresponding author upon reasonable request.

## Abbreviations

CoTCCC: Committee on Tactical Combat Casualty Care
CAT: Combat Application Tourniquet^®^ Generation 7
SAMXT: SAM^®^ Extremity Tourniquet
SOFTT-W: SOF^®^ Tactical Tourniquet Wide Generation 4
